# Sol-Gel-Synthesis of Nanoscopic Complex Metal Fluorides

**DOI:** 10.3390/nano7110362

**Published:** 2017-11-02

**Authors:** Alexander Rehmer, Kerstin Scheurell, Gudrun Scholz, Erhard Kemnitz

**Affiliations:** 1 Institut für Chemie, Humboldt-Universität zu Berlin, Brook-Taylor- Str. 2, D-12489 Berlin, Germany; alex@daboiarussellii.de (A.R.); scheurek@rz.hu-berlin.de (K.S.); gudrun.scholz@chemie.hu-berlin.de (G.S.); 2 Nanofluor GmbH, Rudower Chaussee 29, D-12489 Berlin, Germany

**Keywords:** sol-gel, ternary metal fluorides, nano, ^19^F NMR, XRD

## Abstract

The fluorolytic sol-gel synthesis for binary metal fluorides (AlF_3_, CaF_2_, MgF_2_) has been extended to ternary and quaternary alkaline earth metal fluorides (CaAlF_5_, Ca_2_AlF_7_, LiMgAlF_6_). The formation and crystallization of nanoscopic ternary CaAlF_5_ and Ca_2_AlF_7_ sols in ethanol were studied by ^19^F liquid and solid state NMR (nuclear magnetic resonance) spectroscopy, as well as transmission electron microscopy (TEM). The crystalline phases of the annealed CaAlF_5_, Ca_2_AlF_7_, and LiMgAlF_6_ xerogels between 500 and 700 °C could be determined by X-ray powder diffraction (XRD) and ^19^F solid state NMR spectroscopy. The thermal behavior of un-annealed nanoscopic ternary and quaternary metal fluoride xerogels was ascertained by thermal analysis (TG/DTA). The obtained crystalline phases of CaAlF_5_ and Ca_2_AlF_7_ derived from non-aqueous sol-gel process were compared to crystalline phases from the literature. The corresponding nanoscopic complex metal fluoride could provide a new approach in ceramic and luminescence applications.

## 1. Introduction

The interest in complex metal fluorides (KMgF_3_, CaAlF_5_, SrAlF_5_, LiCaAlF_6_, LiSrAlF_6_, and LiMgAlF_6_) is mainly caused by their thermoluminescent and chemical properties. These complex metal fluorides are often used as host materials for a wide range of applications in the optical luminescence field. They can be applied, for instance, as laser materials, thermoluminescent detectors, or phosphors for lamps and displays [1,2,3,4,5,6,7]. Furthermore, complex fluorides are appropriated in the ceramic field because of their good physical properties like high ionic strength, piezoelectric characteristics, and nonmagnetic insulator behavior [8]. Notably, the ternary metal fluorides (CaAlF_5_, Ca_2_AlF_7_, SrAlF_5_, and Sr_2_AlF_7_) are used as fluoroaluminate substrates due to their very good chemical stability and remarkable optical properties [9,10,11,12]. LiCaAlF_6_, LiMgAlF_6_, and LiSrAlF_6_ are suitable hosts for solid-state lasers [2].

Generally, the synthesis of complex metal fluorides is predominantly based on solid state reactions at high temperatures and/or high pressures or by solvothermal synthesis [13]. The obtained particles usually exhibit diameters in the micrometer range. On the other hand, the fluorolytic sol-gel synthesis, discovered a few years ago, provides in general very convenient and easy access to nanoscopic fluorides [14]. Unfortunately, so far, this synthesis approach has not been deeply investigated for its applicability for the synthesis of complex fluorometallates. Therefore, it was the intention of this work to find out whether or not the applicability of the fluorolytic sol-gel approach can be extended to complex metal fluorides. As we will outline, this non-aqueous fluorolytic sol-gel approach seems to be a universal synthesis route.

Thus, we present an easy room temperature synthesis route to obtain nanoscopic complex metal fluorides which, as a result of thermal treatment between 500 and 700 °C for two minutes, transform into microcrystalline ternary and quaternary metal fluoride compounds. The synthesis of alkali and alkaline earth metal complex fluorides such as KAlF_4_, Na_3_AlF_6_, BaAlF_5_, BaMgF_4_, and K_2_MgF_4_ was successfully developed via the non-aqueous fluorolytic sol-gel route [15,16]. It has to be noted that the synthesis of ternary transition metal fluoride Li_3_MF_6_ (M = transition metal) has also been performed by the non-aqueous fluorolytic sol-gel route [16]. In this work, we adapted the metal chloride-based approach for the synthesis of transparent and stable MgF_2_ sols [17] on complex metal fluorides (CaAlF_5_, Ca_2_AlF_7_, LiMgAlF_6_) because of their good solubility and the practical handling of CaCl_2_ and MgCl_2_ at ambient conditions. Thus, the chloride approach turned out to be a quite robust method compared to the alkoxide-based sol-gel route and enables transparent and long-time stable metal fluoride sols.

## 2. Results and Discussion

The fluorolytic sol-gel synthesis of binary metal fluorides (HS-AlF_3_, MgF_2_, and CaF_2_) derived from different metal precursors such as metal alkoxides, [18,19] metal acetates [20], and metal chlorides [21,22] allows access to monodisperse nanoscopic particles with a broad range of potential applications including optic, catalysis, and ceramic [23]. Notably, we succeeded in the synthesis of transparent and long-time stable CaF_2_ sols prepared from calcium ethoxide and calcium isopropoxide for the first time just by increasing the polarity of the solvent in the CaF_2_ system. We used ethylene glycol instead of methanol or ethanol as the solvent. Thus, the calcium alkoxides (Ca(OEt)_2_ and Ca(O*^i^*Pr)_2_) are soluble at room temperature [24]. We assume that the long-time sol stability of the CaF_2_ nanoparticles could be due to the higher viscosity and polarity of ethylene glycol compared to methanol and ethanol, respectively. 

Due to the access of nanoscaled binary metal fluorides via the fluorolytic sol-gel route, we have adapted the synthesis principle for nanoscopic ternary and quaternary alkaline earth metal fluorides, e.g., by following the chloride approach as developed for MgF_2_ sols [17]. The general synthesis of complex metal fluorides via the chloride approach is represented by Equations (1)–(4).

**Ternary metal fluorides**

(1) $${x\;\mathrm{CaCl}}_{2}+y\;\mathrm{Al}{\left(O^{i}\mathrm{Pr}\right)}_{3}\;\overset{\left(x+y\right){\mathrm{HF}}_{\mathrm{EtOH}}}{\to }{\;\mathrm{nano}\;\mathrm{Ca}}_{x}{\mathrm{Al}}_{y}F_{\left(x+y\right)}+2x\;\mathrm{HCl}+3{y\;}^{i}\mathrm{PrOH}$$

(2) $$\mathrm{LiOMe}+{\mathrm{MgCl}}_{2}\;\overset{{\mathrm{HF}}_{\mathrm{EtOH}}}{\to }\;\mathrm{nano}\;“{\mathrm{LiMgF}}_{3}”\;+2\;\mathrm{HCl}+\mathrm{MeOH}$$

**Quaternary metal fluorides**

(3) LiOMe+Al(OiPr)3+MgCl2 →HFEtOH nano LiMgAlF6+2 HCl+MeOH+3 PirOH

(4) Ba(OH)2+Al(OiPr)3+CaCl2 →HFEtOH nano BaCaAlF7+2 HCl+EtOH+3 PirOH

By using Al(O*^i^*Pr)_3_, CaCl_2_, and EtOH, the formation of transparent ternary calcium fluoroaluminate sols like CaAlF_5_ and Ca_2_AlF_7_ is favored. Further complex metal fluorides such as LiMgAlF_6_, BaCaAlF_7_, and LiMgF_3_ were investigated. It turned out that a stoichiometric composition of LiMgAlF_6_ and LiMgF_3_ resulted in transparent sols (see Figure S1, Supplementary Material). Transparent sols of nanoscopic BaCaAlF_7_ were not obtained under all conditions tested. In the case of LiMgF_3_, there was no indication for its formation under either reaction conditions. Almost nanoscopic MgF_2_ particles, as well as a magnesium alkoxide fluoride species like MgF_2−*x*_OR*_x_* (R = H, Et) [25], were identified by liquid ^19^F NMR spectroscopy of the sol and XRD of the dried xerogel (Figures S2 and S4, supplementary material), which is probably caused by a preferential oxygen donation. Interestingly, the XRD pattern of the annealed nominal LiMgF_3_ compound revealed unidentified reflections in addition to LiF and MgF_2_ (Figure S5, Supplementary Material). Furthermore, the determination of the particle size distribution of all complex metal fluorides by dynamic light scattering (DLS) failed. Although no turbidity of the corresponding sols was observed, there are either invisible agglomerates of primary nanoparticles disturbing the measurements or the very low refractive index of these compounds limits the application of DLS under these circumstances. Even the presence of non-spherical nanoparticles might cause the inappropriateness of DLS for particle size distribution.

### 2.1. CaAlF_5_

The CaF_2_-AlF_3_ binary system is well investigated [26,27]. The first study of CaF_2_-AlF_3_ was described by Fedotiev and Ylinskii [28]. A simple eutectic diagram with an invariant eutectic point at 820 °C and 37.5 mol% AlF_3_ was suggested. There are two compounds in the binary CaF_2_-AlF_3_ system; a dimorphic CaAlF_5_ which has a reversible α ↔ β transition around 740 °C and Ca_2_AlF_7_.

CaAlF_5_ was first identified by Holm by differential thermal analysis (DTA) experiments [29]. It melts incongruently at 873 °C ± 3 °C to form AlF_3_ and an un-identified liquid phase. Powder diffraction measurements and ^19^F MAS NMR spectroscopy were performed to confirm the crystal structure of CaAlF_5_ [30,31,32] and Ca_2_AlF_7_ [32,33,34,35]. Both crystal structures correspond to those found in the naturally occurring phases in *Jakobssonite* [36] (CaAlF_5_) and *Carlhintzeite* [37] (Ca_2_AlF_7_∙H_2_O), respectively. However, to the best of our knowledge, no synthetic phase of CaAl_2_F_8_, which occurs in *Prosopite* (CaAl_2_(F,OH)_8_), has been reported so far.

**Figure 1:**
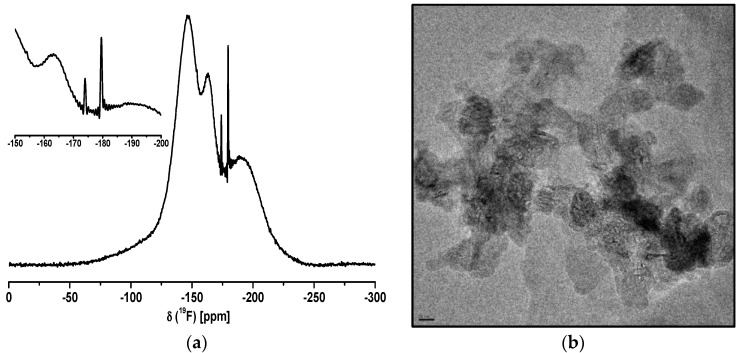
^19^F liquid NMR spectrum (**a**) and TEM image (**b**) CaAlF_5_ sol in EtOH (scale bar = 20 nm).

In Figure 1, the ^19^F liquid NMR spectrum of CaAlF_5_ sol is shown. Due to the formation of nanoparticles in the sol, the line width of the NMR signals for CaAlF_5_ species is very broad. The crystal structure of α-CaAlF_5_ reveals three ^19^F NMR peaks which correspond to three distinct fluorine sites with multiplicities of 2, 2, and 1. The fluorine sites in CaAlF_5_ are connected with one aluminum and two calcium Al-F-Ca(2), one aluminum and calcium Al-F-Ca, and between two aluminum and one calcium Al-F-Al(+Ca), with chemical shifts relative to CF_3_Cl of −146.2, −154.3, and −164 ppm, respectively [32,35]. Furthermore, the liquid NMR spectrum of the CaAlF_5_ sol shows two small signals (line width around 350 Hz) at −174 ppm and −179 ppm, which can probably be assigned to unreacted HF adsorbed at the particle surface in a mix of isopropanol and ethanol [21,22,38]. The presence of isopropanol in the CaAlF_5_ sol is probably caused by the reaction of Al(O*^i^*Pr)_3_ with HF-solution. The broad main signals at about −146 and −164 ppm in the liquid NMR spectrum can be indicated as known species of CaAlF_5_. A ^19^F NMR signal for CaF_2_ (−108 ppm) in the spectrum could not be observed. The broad signal around −190 ppm is the known background from the fluorine NMR probe. 

**Figure 2:**
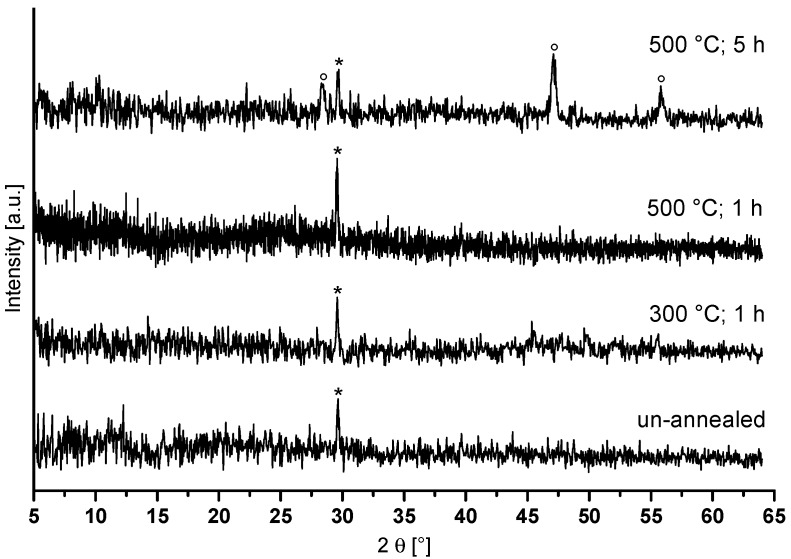
Powder diffractograms of CaAlF_5_ xerogel in a normal crucible at different thermal treatment in a non-preheating oven (* sample holder, ° CaF_2_).

**Figure 3:**
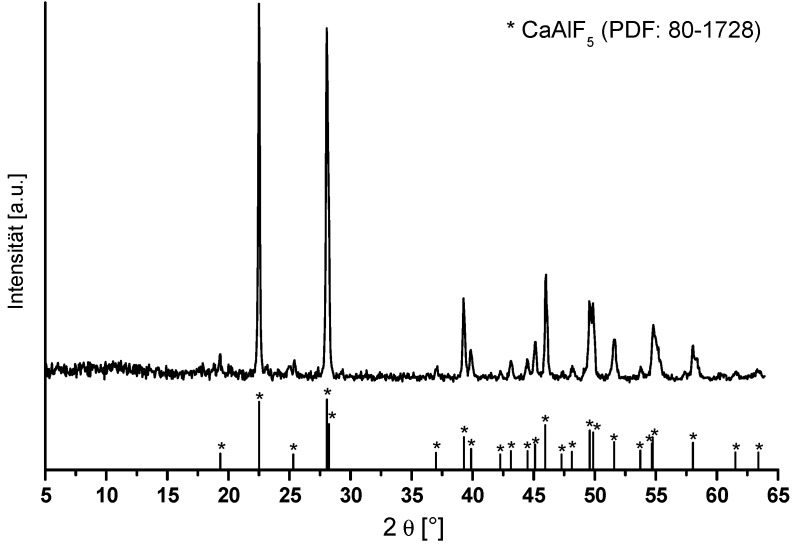
Comparison of powder X-ray patterns of CaAlF_5_ xerogel in a closed crucible at 700 °C for a two minute calcination time in a preheated oven at 700 °C with crystalline CaAlF_5_ phase (PDF: 80–1728).

Due to the problem of the particle size characterization by DLS, the particle size and shape were investigated by TEM. Figure 1b shows a group of several agglomerated particles showing diameters above 20 nm, but it seems that the particles exhibit a nearly spherical shape. After evaporation of the solvent from CaAlF_5_ sol, the CaAlF_5_ xerogel was characterized by XRD and ^19^F MAS NMR spectroscopy. Calcination below 500 °C revealed still X-ray amorphous samples. Samples calcined at 500 °C for 5 h gave reflections of CaF_2_ as indicated in the diffractogram (Figure 2). No X-ray patterns of CaAlF_5_ could be identified. Interestingly, if the xerogel was annealed at 700 °C for only a two minute calcination time in a closed crucible, the reflections of CaAlF_5_ can be observed (Figure 3). It is crucial to determine whether an open or closed crucible and whether a preheated or non-preheated oven is used. Probably, the calcination process of CaAlF_5_ results in a sublimation of AlF_3_. This could explain the formation of CaF_2_ in the CaAlF_5_ xerogel after calcination at 500 °C for 2 h.

**Figure 4:**
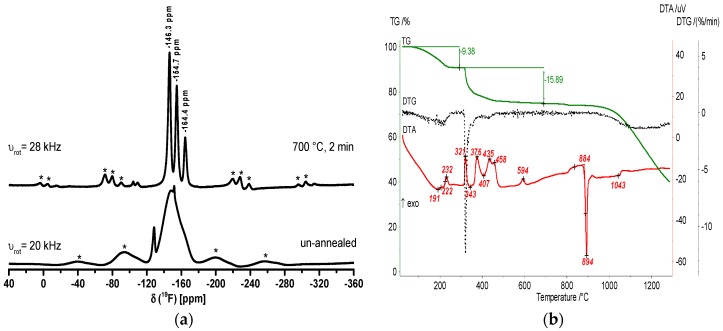
^19^F MAS NMR spectra of un-annealed and annealed CaAlF_5_ xerogel at 700 °C for two minutes (**a**) and TG/DTA heating curves (**b**). The spinning sidebands in the MAS NMR spectrum are located under the * symbols.

In Figure 4, the ^19^F MAS NMR spectra and the TG/DTA heating curves of CaAlF_5_ xerogel confirm the formation of crystalline CaAlF_5_. The spectrum of the un-annealed xerogel exhibits one broad signal around −149 ppm and two small signals (−128 ppm and −152 ppm). Both small signals can be indicated as SiF_6_^2−^ (−128 ppm) and BF_4_^−^ (−152 ppm) species, which result from the unreacted HF with the glass flask. It is obvious that the thermal treatment of the xerogel at 700 °C reveals three signals in the ^19^F NMR spectrum, which correspond to crystalline α-CaAlF_5_ with a relative intensity of 45, 36, and 18. In the literature, α-CaAlF_5_ has three ^19^F signals with a relative intensity of 40, 40, and 20, whereas β-CaAlF_5_ has four ^19^F signals with a relative intensity of 15, 57, 15, and 13 [32]. Thus, we think that the annealed xerogel correlates more α-CaAlF_5_ than β-CaAlF_5_ phase. Especially, the phase diagram of the CaF_2_-AlF_3_ binary system with 50 mol% CaF_2_ confirms the presence of α-CaAlF_5_ until 743 °C ± 3 °C [26]. Furthermore, the thermal behavior of the CaAlF_5_ xerogel was investigated by thermogravimetry and DTA. The DTA heating curve of the CaAlF_5_ xerogel shows several exothermic peaks below 600 °C and a melting point at 894 °C. Craig and Brown found a melting point for β-CaAlF_5_ at 879 °C in the CaF_2_-AlF_3_ binary system [26]. Due to the synthesis in ethanol and the aluminum alkoxide precursor, the organic residue of ethanol in the xerogel is still present. Thus, the peak at 321 °C is probably the decomposition of the organic residue. At around 458 °C, we assume that the polymorphic inversion of AlF_3_ occurs (≈454 °C), which is noted by Holm [39]. 

### 2.2. Ca_2_AlF_7_

**Figure 5:**
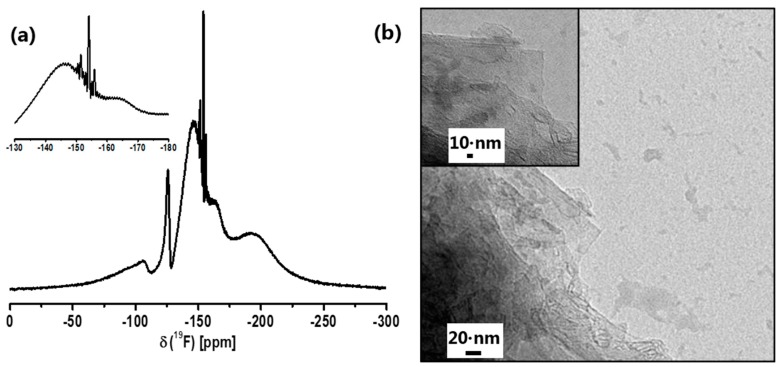
^19^F liquid NMR spectrum (**a**) and TEM image (**b**) of Ca_2_AlF_7_ sol in EtOH.

In the Ca_2_AlF_7_ system, we only obtained a transparent and long-time stable sol by the addition of 5 mol% TMOS (tetramethyl orthosilicate). Apparently, the amount of 66 mol% Cl instead of 50 mol% Cl results in a faster agglomeration of the particles followed by sedimentation. According to pure CaF_2_ sols from CaCl_2_ in EtOH, we observed the same effect of particle agglomeration. The addition of 5 mol% TMOS to an opaque CaF_2_ sol that was formed after HF addition resulted in a transparent sol that was obtained within a few minutes [22]. Hence, the chloride amount in the calcium fluoroaluminate system is crucial to determing whether a transparent or turbid sol is formed. ^19^F liquid NMR spectrum and TEM images of Ca_2_AlF_7_ sol are shown in Figure 5.

In the ^19^F liquid NMR spectrum of the Ca_2_AlF_7_ sol, the corresponding broad signals at around −146 ppm and −163 ppm can be assigned to Ca_2_AlF_7_ and CaAlF_5_, respectively. Moreover, the signals at −106 and −125 ppm are characteristic for CaF_2_ and SiF_6_^2−^-species. The small signals between −150 and −156 ppm are related to alkoxyfluorosilanes (RO)_4−*x*_-SiF*_x_* (R = Me, Et). [40] The crystal structure of Ca_2_AlF_7_ has five distinct fluorine sites. One fluorine site is connected to three calcium F-Ca(3) and four fluorine sites are connected to aluminum and calcium with varying numbers of calcium Al-F-Ca(n) [33]. The corresponding ^19^F MAS NMR spectrum of Ca_2_AlF_7_ reveals three signals with chemical shifts relative to CF_3_Cl of −104.0, −146.7, and −152.2 ppm [35]. 

The TEM image of the Ca_2_AlF_7_ sol represents small spherical-like particles. Remarkably, the agglomeration partially leads to an angular shape of bigger particles, which is shown in the insight of the TEM image.

**Figure 6:**
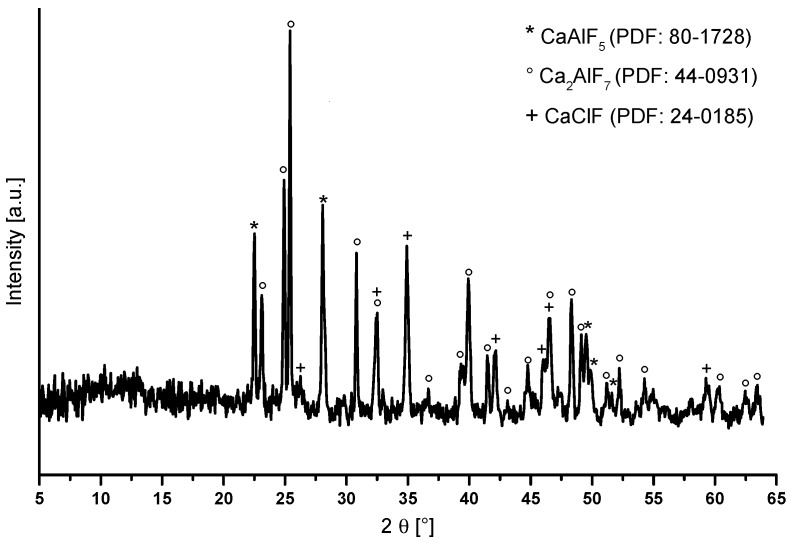
Powder diffractogram of annealed Ca_2_AlF_7_ xerogel at 700 °C for two minutes with reflections of Ca_2_AlF_7_, CaAlF_5_, and CaClF.

The powder diffractogram of the annealed Ca_2_AlF_7_ xerogel shows reflections of Ca_2_AlF_7_, CaAlF_5_, and CaClF (Figure 6). The phase diagram of CaF_2_-AlF_3_ with a composition of 66 mol% CaF_2_ predicts CaAlF_5_ and Ca_2_AlF_7_. The formation of CaClF was already reported and discussed in the synthesis of CaF_2_ sols derived from CaCl_2_ [22].

**Figure 7:**
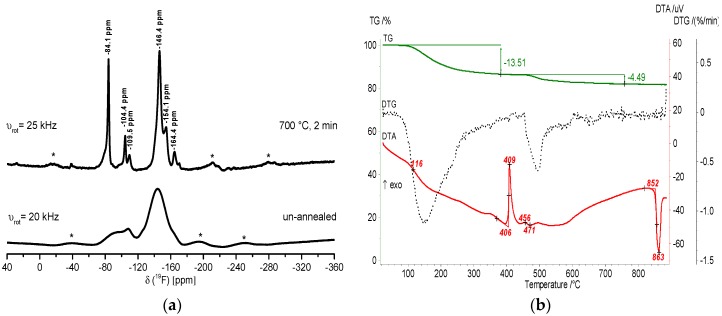
^19^F MAS spectra of un-annealed and annealed Ca_2_AlF_7_ xerogel at 700 °C for two minutes (**a**) and TG/DTA heating curves (**b**). The spinning sidebands in the NMR spectrum are located under the * symbols.

In Figure 7, the recorded ^19^F MAS NMR spectra of an un-annealed and annealed xerogel and the corresponding TG/DTA heating curves confirm the formation of crystalline Ca_2_AlF_7_. In the NMR spectrum of the un-annealed sample, there are two very broad signals between −70 and −110 ppm and −120 and −170 ppm. After calcination of the Ca_2_AlF_7_ xerogel at 700 °C, the spectrum represents three signals at −104.4, −146.4, and −154.1 ppm, which stand for Ca_2_AlF_7_. The signals at −84.1, −109.5, and −164.4 ppm correspond to CaClF, CaF_2_, and CaAlF_5_, respectively.

The DTA curve of the Ca_2_AlF_7_ xerogel shows one exothermic peak at 409 °C. We assume that the crystallization of CaClF occurred at this temperature. The peak at 863 °C corresponds to the melting point of Ca_2_AlF_7_, which is lower than the melting point of the CaAlF_5_ xerogel at 893 °C. The temperature difference of both melting points is approximately 30 °C. Thus, the melting point of Ca_2_AlF_7_ in the phase diagram of the CaF_2_-AlF_3_ binary system is 850 °C. The temperature difference of both melting points from the literature and that of our investigations reveals nearly the same temperature difference of 30 °C. Hence, we are convinced that the calcium fluoroaluminates synthesized via the fluorolytic sol-gel route exhibit a similar thermal behavior to microcrystalline calcium fluoroaluminates. Therefore, we also investigated the third calcium fluoroaluminate compound CaAl_2_F_8_ with a metal content of 66 mol% Al, which is discussed in the next section.

### 2.3. CaAl_2_F_8_

**Figure 8:**
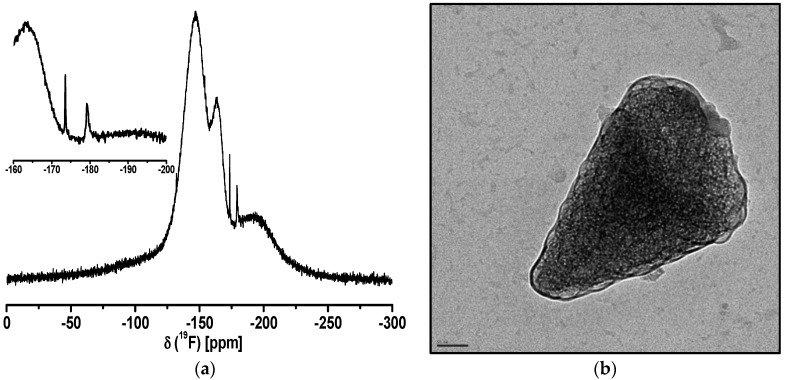
^19^F liquid NMR spectrum (**a**) and TEM image (**b**) of the nominal “CaAl_2_F_8_” sol in EtOH (scale bar = 50 nm).

Although there are no reports on the synthetic calcium fluoroaluminate, CaAl_2_F_8_, we prepared a sol with a nominal stoichiometry of CaAl_2_F_8_. A water clear sol was obtained in this case without the addition of TMOS. In Figure 8, the ^19^F liquid NMR spectrum of the nominal “CaAl_2_F_8_” sol resembles the spectrum of the CaAlF_5_ sol (Figure 1). There are two broad and two small signals. The two broad signals at around −147 and −163 ppm correspond to CaAlF_5_ and the two signals at −173 and −179 ppm could probably be assigned to unreacted HF adsorbed at the particle surface. The particle size is about 10–20 nm. But the particles have a rather non-spherical shape and tend to agglomerate to form big secondary particles of a 200 nm diameter.

**Figure 9:**
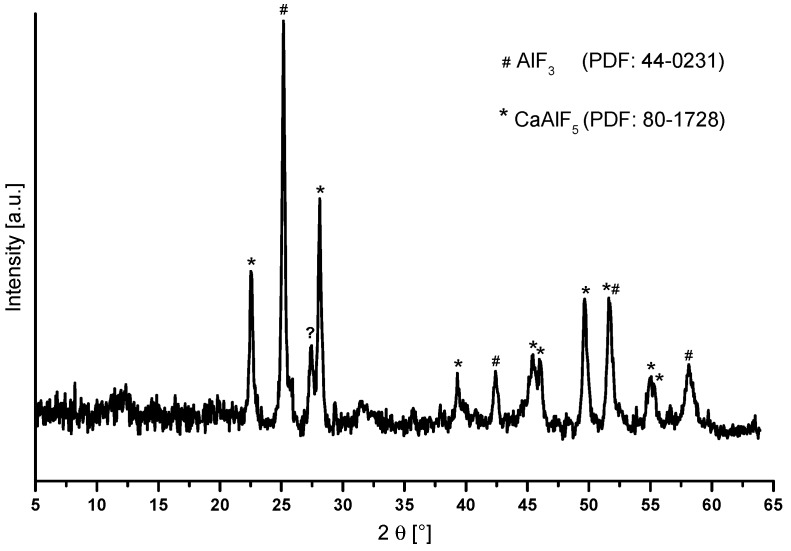
Powder diffractogram of the annealed nominal “CaAl_2_F_8_” xerogel at 700 °C for two minutes with reflections of CaAlF_5_ and AlF_3_.

The annealed “CaAl_2_F_8_” xerogel was further investigated by powder X-ray diffraction. Figure 9 shows the recorded diffractogram of the “CaAl_2_F_8_” xerogel. There are reflections of CaAlF_5_ and AlF_3_, and a reflection that cannot be assigned to any known phase. It is obvious that no “CaAl_2_F_8_” phase has been formed under these conditions. This is also in agreement with the CaF_2_-AlF_3_ binary phase diagram. At a ratio of 66 mol% Al and 33% mol% Ca at 700 °C, the formation of crystalline α-CaAlF_5_ and β-AlF_3_ is expected.

**Figure 10:**
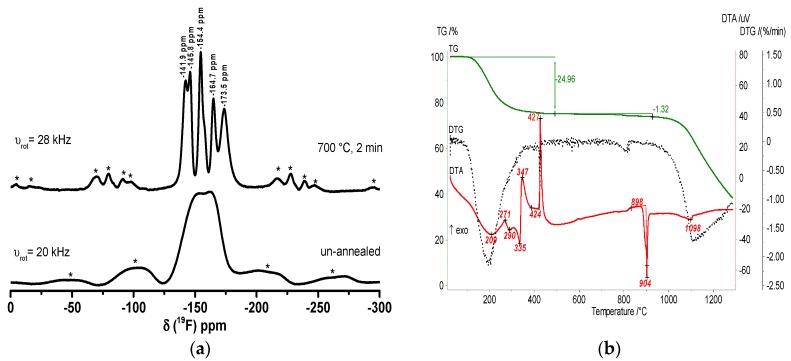
^19^F MAS spectra of un-annealed and annealed CaAl_2_F_8_ xerogel at 700 °C for two minutes (**a**) and TG/DTA heating curves (**b**). The spinning sidebands in the NMR spectrum are located under the * symbols.

In line with this, the ^19^F MAS spectrum of the un-annealed sample reveals one broad signal between −125 and −175 ppm, and the “CaAl_2_F_8_” xerogel after annealing at 700 °C for two minutes in a closed crucible exhibits five signals in the spectrum. Three signals at −145.8, −154.4, and −164.7 ppm stand for CaAlF_5_. The signal at −141.9 ppm can probably be assigned to β-CaAlF_5_, which has four different fluorine sites. The last signal at −173.5 ppm can be assigned to AlF_3_ (Figure 10a). The DTA heating curve of the “CaAl_2_F_8_” xerogel shows two exothermic peaks at 347 and 427 °C, which stand for the crystallization and polymorphic inversion of α-AlF_3_. The peak at 904 °C indicates the melting of CaAlF_5_, which is consistent with the phase diagram of the binary CaF_2_-AlF_3_ system. The “CaAl_2_F_8_” xerogel in fact consists of CaAlF_5_ and AlF_3_ (Figure 10b). This means that no formation of a “CaAl_2_F_8_” phase was detected under these conditions.

### 2.4. LiMgAlF_6_

**Figure 11:**
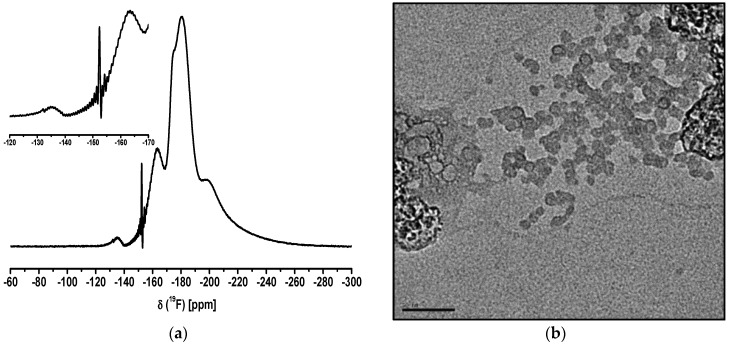
^19^F liquid NMR spectrum (**a**) and TEM image (**b**) of LiMgAlF_6_ sol in EtOH (scale bar = 50 nm).

The possibility to achieve quaternary metal fluorides by fluorolytic sol-gel synthesis was also tested. We achieved transparent and stable LiMgAlF_6_ sols in EtOH. The ^19^F liquid NMR spectrum of the LiMgAlF_6_ sol is shown in Figure 11a and reveals two broad signals at −164 and −182 ppm. The signal at −164 ppm is probably a fluorinated aluminum alkoxide species. The signal at −182 ppm with the corresponding shoulder can either be ascribed to a high amount of unreacted HF adsorbed at the particle surface or incompletely fluorinated MgCl_2_. The small broad signal (−135 ppm) and the small signal (−153 ppm) are due to the reaction of the unreacted HF with the glass flask. Hence, the signals at −135 ppm and −153 ppm are SiF*_x_* and BF_4_^−^ species, respectively. The TEM image in Figure 11b shows nearly spherical shape particles with a size of about 10 nm. 

**Figure 12:**
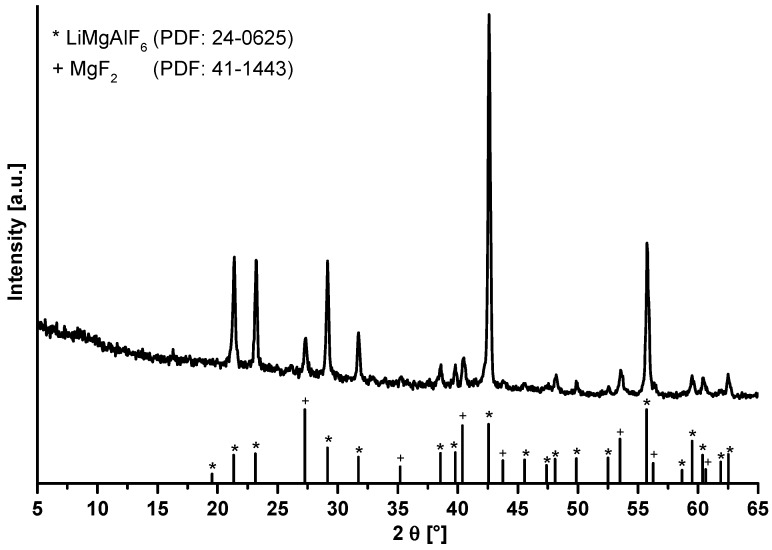
Comparison of powder X-ray patterns of LiMgAlF_6_ xerogel in an open crucible at 500 °C for a one hour calcination time in a non-preheated oven with crystalline LiMgAlF_6_ phase (PDF: 24-0625) and MgF_2_ phase (PDF: 41-1443).

The annealed LiMgAlF_6_ xerogel was measured by powder X-ray diffraction. As can be seen in Figure 12, there are reflections of the LiMgAlF_6_ phase and MgF_2_ in the diffractogram. Surprisingly, the use of a closed crucible is not necessary. It can be assumed that the formation of AlF_3_ is thermodynamically not favored in this reaction system, unlike the formation of MgF_2_.

**Figure 13:**
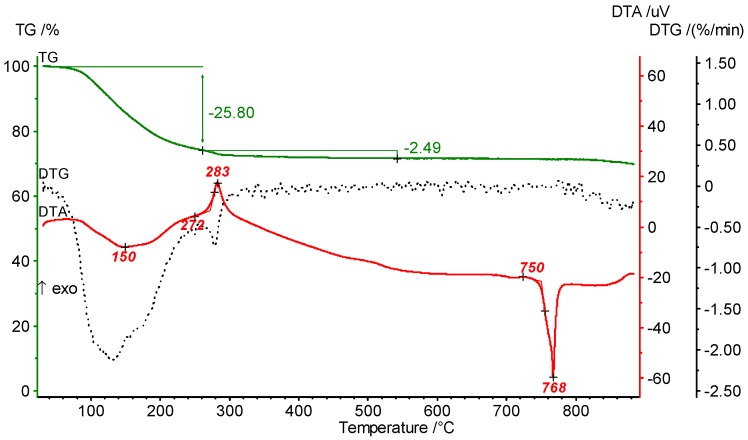
TG/DTA heating curves of LiMgAlF_6_.

The DTA heating curve of the LiMgAlF_6_ xerogel (Figure 13) shows an exothermic peak at 383 °C which stands for crystallization and the peak at 768 °C compares very well to the melting point of LiMgAlF_6_.

## 3. Materials and Methods

### 3.1. Synthesis of Complex Metal Fluoride Sols

All chemicals for the synthesis of ternary and quaternary metal fluorides are commercially available and need no drying or further processing. The 19.05 M HF-solution was prepared by dissolving anhydrous HF in ethanol. The molar concentration of the sols refers to the total metal concentration of 0.4 M in the corresponding complex metal fluoride compound.

The stoichiometric fluoride compounds CaAl_2_F_8_, CaAlF_5_, LiMgF_3_, and LiMgAlF_6_ were prepared as follows: Anhydrous CaCl_2_ (≥97%, Sigma-Aldrich), MgCl_2_ (≥98%, Sigma-Aldrich, Schnelldorf, Germany), and LiOMe (95%, Strem Chemicals, Kehl, Germany), respectively, were dissolved in 50 mL ethanol (99.8%, Roth, Karlsruhe, Germany). Afterwards, Al(O*^i^*Pr)_3_ (≥98%, Sigma-Aldrich) was suspended into the solution. Under vigorous stirring at ambient conditions, the required amount of HF-solution was added dropwise to the suspension. In case of Ca_2_AlF_7_, the addition of 5 mol% tetramethyl orthosilicate (TMOS) after fluorination turns an opaque sol into a transparent Ca_2_AlF_7_ sol. Apparently, the presence of metal chloride in the sol and the addition of TMOS after fluorination ensures a higher electrostatic repulsion of the nanoparticles than without the addition of TMOS which increases the particle stabilization. The sols were dried under vacuum at 80 °C to obtain the corresponding xerogels.

### 3.2. Analytical Measurements

The ^19^F NMR spectra of the sols were obtained by using a Bruker AVANCE II (liquid state NMR spectrometer with a Larmor frequency of 282.4 MHz). The ^19^F isotropic chemical shifts are given with respect to the CFCl_3_ standard. 

Transmission Electron Microscope (TEM) analysis was carried out using a Philips CM200 LaB_6_ microscope operating at 200 kV. A few drops of the solution (0.1 mM) containing the nanoparticles were deposited on a carbon-coated copper grid and were left to dry prior to the inspection.

Thermal analysis experiments of the complex metal fluorides were performed on a STA 409 °C (Netzsch Gerätebau GmbH, Selb, Germany). A DTA-TG sample-holder system (Pt/PtRh10 thermocouple) was used. The thermoanalytical curves (TG, DTA and DTG) were recorded under air atmosphere with a constant heating rate of 10 K/min.

The xerogels were characterized by an X-ray powder diffractometer from Seifert (XRD 3003 TT) and by ^19^F solid state NMR spectroscopy. Phases were identified by a comparison with the ICDD (international center for diffraction data) powder diffraction file [41]. The ^19^F MAS NMR spectra were recorded with a Bruker AVANCE 400 (solid state spectrometer, Larmor frequency of 376.4 MHz) with a π/2 pulse duration of 3.6 μs in a 2.5 mm Bruker probe, a recycle delay of 5 s, and accumulation number of 64. The experiments were performed with a rotation frequency of 20 kHz. The ^19^F isotropic chemical shifts are given with respect to the CFCl_3_ standard. The xerogels were calcined in a preheated vented air-oven (Barnstead thermolyne F47900, Dubuque, IA, USA) at 500 °C and 700 °C, respectively. The dwell time was 2 and 60 min, respectively. After the dwell time, the sample was quickly taken out of the oven and cooled down to room temperature. 

## 4. Conclusions

The fluorolytic sol-gel synthesis, so far mainly used for the synthesis of binary or ternary metal fluorides, has been successfully applied for the synthesis of nanoscaled, homodispersed complex metal fluoride sols (CaAlF_5_, Ca_2_AlF_7_, LiMgAlF_6_) using CaCl_2_, MgCl_2_, LiOMe, and Al(O*^i^*Pr)_3_ as metal precursors, which were reacted with anhydrous HF in ethanol. All complex metal fluoride sols showed long-time stability and transparency even after months. These complex metal fluorides recently gained an enormous amount of interest as hosts for fluorescent materials [1,2,3,4,5,6,7] due to the suitable sizes of the ionic radii of alkaline earth cations. In addition, they exhibit high ionic strength, hardness, good isolation behavior, and are stable over a wide temperature range. Hence, based on the new synthesis approach reported here, an alternative and very effective synthesis approach for such fluorescent materials is provided. Even bulk ceramics are accessible starting from such nano powdered complex metal fluorides. Based on XRD and MAS-NMR-investigations, the formation of these complex metal fluorides already at room temperature was unambiguously proven. Thus, these results show that the fluorolytic sol gel synthesis is not limited to binary metal fluorides, but can also be successfully adapted for even more complex metal fluoride systems.

Thus, a new synthesis path has been explored for a variety of new, optically very interesting complex metal fluoride-based materials.

## Supplementary Materials

The following are available online at www.mdpi.com/2079-4991/7/11/362/s1, Figure S1: Photograph of a typical transparent LiMgF_3_ sol, Figure S2: ^19^F liquid NMR spectrum of LiMgF_3_ sol, Figure S3: TEM image of LiMgF_3_ sol, Figure S4: X-Ray powder diffractogram of un-annealed LiMgF_3_ xerogel, Figure S5: Comparison of X-ray powder patterns of annealed LiMgF_3_ xerogel at 700 °C (a), 850 °C (b) for 2 min and crystalline MgF_2_ (PDF: 41-1443) and LiF (PDF: 04-0857), Figure S6: TG/DTA heating curves of LiMgF_3_ xerogel.
