# Supplementary material for: Sol-Gel-Synthesis of Nanoscopic Complex Metal Fluorides
Source: Nanomaterials (Basel). 2017 Nov 2;7(11):362. doi: 10.3390/nano7110362 (PMC5707579; doi:10.3390/nano7110362)
Supplement: Supplementary file 1 [file nanomaterials-07-00362-s001.pdf]

Sol-Gel-Synthesis of Nanoscopic Complex Metal Fluorides

Alexander Rehmer [1], Kerstin Scheurell [1], Gudrun Scholz [1] and Erhard Kemnitz [1,2,*]

Supplementary material

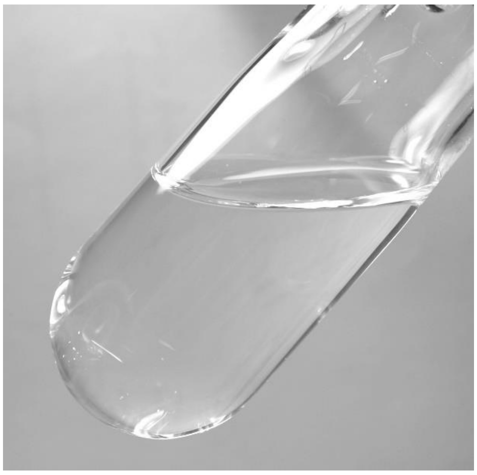

Figure S1. Photograph of a typical transparent $LiMgF_3$ sol.

"$LiMgF_3$"

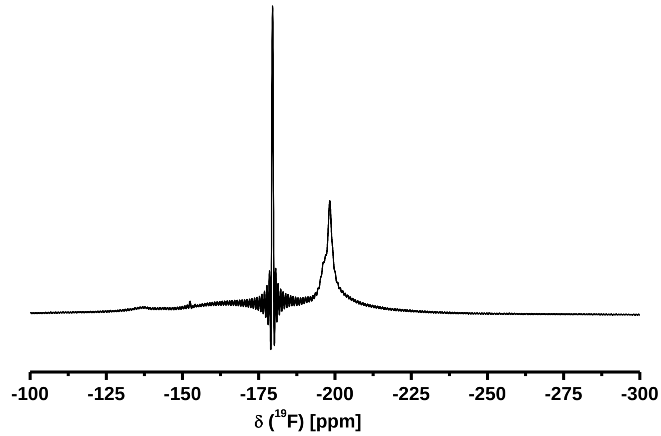

Figure S2. ^{19}F liquid NMR spectrum of $LiMgF_3$ sol.

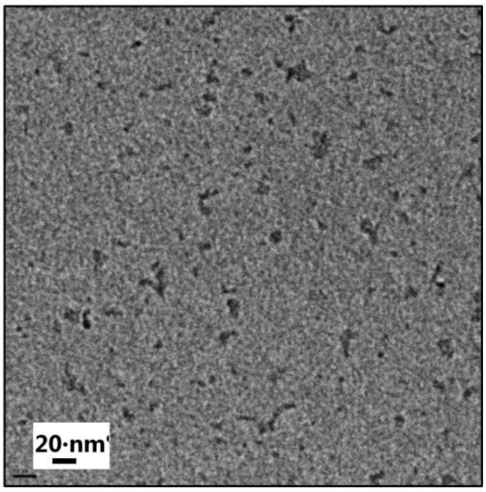

Figure S3. TEM image of $LiMgF_3$ sol.

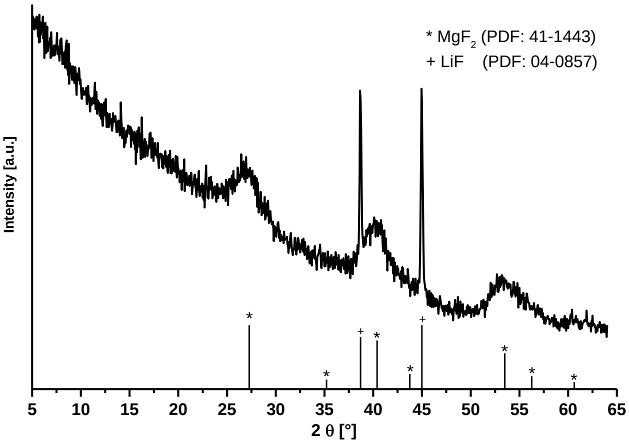

Figure S4. X-Ray powder diffractogram of un-annealed $LiMgF_3$ xerogel.

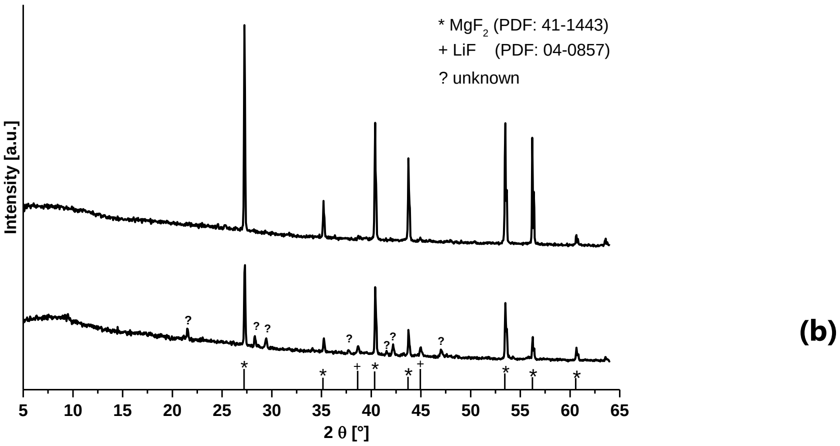

Figure S5. Comparison of X-ray powder patterns of annealed $LiMgF_3$ xerogel at 700 °C (a), 850 °(a)b) for 2 minutes and crystalline MgF_2 (PDF: 41-1443) and LiF (PDF: 04-0857).

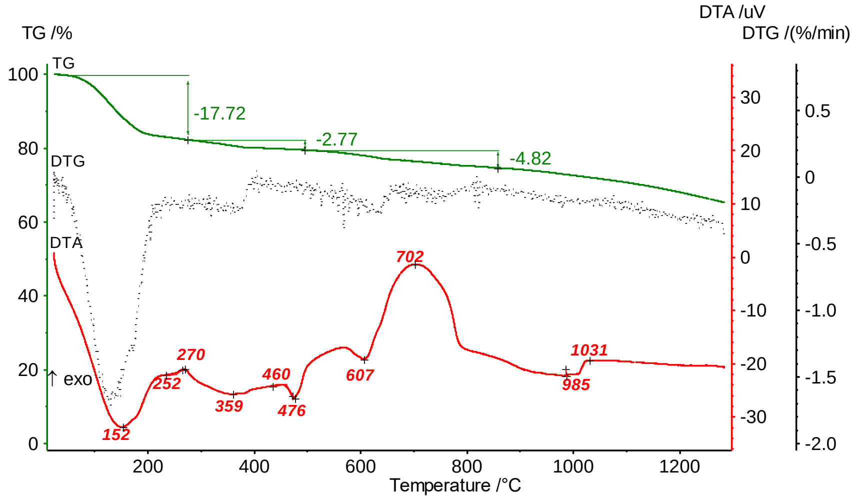

Figure S6. TG/DTA heating curves of $LiMgF_3$ xerogel.
